# Tissue- and Sex-Specific DNA Methylation Changes in Mice Perinatally Exposed to Lead (Pb)

**DOI:** 10.3389/fgene.2020.00840

**Published:** 2020-08-21

**Authors:** Kai Wang, Siyu Liu, Laurie K. Svoboda, Christine A. Rygiel, Kari Neier, Tamara R. Jones, Justin A. Colacino, Dana C. Dolinoy, Maureen A. Sartor

**Affiliations:** ^1^Department of Computational Medicine and Bioinformatics, School of Medicine, University of Michigan, Ann Arbor, MI, United States; ^2^Department of Environmental Health Sciences, School of Public Health, University of Michigan, Ann Arbor, MI, United States; ^3^Department of Biostatistics, School of Public Health, University of Michigan, Ann Arbor, MI, United States

**Keywords:** DNA methylation, lead exposure, ERRBS, tissue, sex

## Abstract

Lead (Pb) is a well-known toxicant that interferes with the development of a child’s nervous and metabolic systems and increases the risk of developing diseases later in life. Although studies have investigated epigenetic effects associated with Pb exposure, knowledge of genome-wide changes with *in vivo* low dose perinatal Pb exposure in multiple tissues is limited. Within the Toxicant Exposures and Responses by Genomic and Epigenomic Regulators of Transcription (TaRGET II) consortium, we utilized a mouse model to investigate tissue- and sex-specific DNA methylation. Dams were assigned to control or Pb-acetate water, respectively. Exposures started 2 weeks prior to mating and continued until weaning at post-natal day 21 (PND21). Liver and blood were collected from PND21 mice, and the DNA methylome was assessed using enhanced reduced representation bisulfite sequencing (ERRBS). We identified ∼1000 perinatal Pb exposure related differentially methylated cytosines (DMCs) for each tissue- and sex-specific comparison, and hundreds of tissue- and sex-specific differentially methylated regions (DMRs). Several mouse imprinted genes were differentially methylated across both tissues in males and females. Overall, our findings demonstrate that perinatal Pb exposure can induce tissue- and sex-specific DNA methylation changes and provide information for future Pb studies in humans.

## Introduction

DNA methylation is a chemical modification of DNA that can affect gene expression without changing the underlying sequence (Robertson, 2005). During embryonic development of mammals, DNA methylation from parents is for the most part erased and re-established in gametogenesis and early embryogenesis (McSwiggin and O’Doherty, 2018). DNA methyltransferases (DNMTs) are required for establishment and maintenance of DNA methylation (Castillo-Aguilera et al., 2017). Another family of enzymes, ten-eleven translocation (TET), is involved in the demethylation process in mammals (Kohli and Zhang, 2013). High levels of DNA methylation in gene promoter regions are associated with repression of transcriptional activity (Weber et al., 2007), and DNA methylation changes are associated with many diseases, including cancer, atherosclerosis, and Alzheimer’s disease (Zawia et al., 2009; Bergman and Cedar, 2013). A variety of environmental exposures, including air pollution, tobacco smoke, and toxicants, including metals, are associated with altered DNA methylation levels (Baccarelli et al., 2009; Fragou et al., 2011). Reprogramed DNA methylation levels during development can be maintained through cell division and influence disease status in later life. Therefore, it is important to investigate the DNA methylation changes resulting from early life exposures (Ideta-Otsuka et al., 2017).

Lead (Pb) is a well-known toxicant that affects almost all human organs and systems (Muller et al., 2018). In the United States, approximately 400,000 deaths every year have been attributed to Pb exposure (Lanphear et al., 2018). Even low blood Pb levels (<5 μg/dL) are associated with increased risk of certain diseases such as hypertension, atherosclerosis, and left-ventricular hypertrophy. Children with Pb exposure are at increased risk of developing non-communicable chronic diseases in later life (Lanphear et al., 2018), and children with blood Pb levels <7.5 μg/dL demonstrate intellectual deficits (Lanphear et al., 2019). Recent studies show that Pb can influence DNA methylation, which can further impair cognitive development and result in behavioral problems (Min et al., 2017). Besides the effects of Pb on the nervous system, Pb can also accumulate in and cause oxidative damage to many human tissues, including heart, liver, kidney, and reproductive organs (Patra et al., 2001; Long et al., 2016). At the cellular level, Pb enhances the peroxidation of membrane lipids, affects membrane proteins and ultimately damages the molecular functions (Sandhir and Gill, 1995). In mouse brain, Pb induced DNA methylation changes are correlated with altered mRNA expression (Sánchez-Martín et al., 2015). In addition to coding genes, DNA methylation status of murine IAP transposons can be influenced by Pb exposure (Montrose et al., 2017). Although studies exist on the epigenetic effects of Pb exposure, research on environmentally relevant perinatal Pb exposure in multiple tissues within the same cohort are limited.

To understand how environmentally relevant perinatal Pb exposure affects genome-wide DNA methylation in different tissues, we performed studies as part of the Toxicant Exposures and Responses by Genomic and Epigenomic Regulators of Transcription (TaRGET II) consortium (Wang et al., 2018). In this study, a mouse model of human-relevant environmental exposure to Pb was used to investigate tissue- and sex-specific DNA methylation. Enhanced reduced representation bisulfite sequencing (ERRBS) (Garrett-Bakelman et al., 2015) was used to measure DNA methylation changes in liver (target tissue) and blood (surrogate tissue) collected from post-natal day 21 (PND21) mice with and without perinatal Pb exposure.

## Materials and Methods

### Animal Exposure, DNA Extraction, and ERRBS

In this study, experiments were performed in wild-type non-agouti *a/a* mice from a colony of viable yellow agouti (*A*^*vy*^) mice maintained over 230 generations resulting in mice genetically invariant that are 93% identical to C57BL/6J (Waterland and Jirtle, 2003; Weinhouse et al., 2014). Exposure to Pb was carried out by adding Pb-acetate to drinking water, which was provided to mice *ad libitum*. Pb-acetate mixed with distilled water with a Pb concentration of 32 ppm was used for exposure. This concentration results in human relevant maternal exposure levels in the 16–60 μg/dL range, as previously described (Faulk et al., 2013). Dams were randomly assigned to control and exposure groups. The exposure started 2 weeks prior to mating and continued until weaning at post-natal day 21 (PND21). Tissues (i.e., blood and liver) were collected from 7 males and 7 females for each group, 1–2 males and 1–2 females per litter. Blood was collected through cardiac puncture immediately following CO2 euthanasia and the left lobe of the liver was dissected and flash frozen in liquid nitrogen and then stored at −80°C. AllPrep DNA/RNA/miRNA Universal Kit (Qiagen #80224) was used for DNA extractions. Enhanced reduced representation bisulfite sequencing was performed at the University of Michigan Epigenomics and Advanced Genomics Cores (Garrett-Bakelman et al., 2015). More details about animal exposure, tissue collection, DNA extraction, and ERRBS can be found in Svoboda et al. (2019). Procedures of this study were approved by the University of Michigan Institutional Animal Care and Use Committee (IACUC).

### Data Processing and DMC/DMR Analysis

For quality control of ERRBS data, FastQC (v0.11.3) was used to assess the quality of all sequenced samples (Andrews, 2010). TrimGalore (v0.4.5) was applied to trim adapter and low quality bases (Krueger, 2015). After trimming, reads shorter than 20 bp were removed from further analysis. Bismark (v0.19.0) was used for mapping and methylation calling (Krueger and Andrews, 2011) with Genome Reference Consortium Mouse Build 38 (mm10) as the reference genome. Bowtie2 (v2.3.4) was used as backend alignment software, and all alignments were performed with default parameters, i.e., 0 mismatches with multi-seed length of 20 bp (Langmead and Salzberg, 2012). The unmethylated lambda phage DNA was used to calculate the bisulfite conversion rates. For methylation calls, CpG sites with less than five reads covered were discarded from further analysis. The wCorr R package (version 1.9.1) was used to calculate the weighted genome-wide pair-wise sample correlations of CpG methylation, with the read counts at each CpG site as the weights. The methylSig R package (version 0.5.2) was used to detect differentially methylated cytosines (DMCs) and differentially methylated regions (DMRs) (Park et al., 2014). A window size of 50 bp was applied for discovering DMRs. The differential methylation test was performed by using the *methylSigDSS* function. Cytosines or windows with sufficient coverage in at least four samples per treatment were used for DMC and DMR detection, respectively. Adjusted *p*-values were obtained using FDR (Benjamini and Hochberg, 1995). FDR less/equal than 0.15 and absolute difference in methylation larger/equal than 10% were used to obtain the final DMCs and DMRs. Significant DMCs/DMRs were annotated with the annotatr Bioconductor package (version 1.8.0) (Cavalcante and Sartor, 2017). The *annotate_region* function was used to generate different genomic annotations, including CpG islands, CpG shores, CpG shelves, CpG intervals, promoters, exons, introns, 5′UTRs, 3′UTRs, enhancers, and 1–5 kb upstream of TSSs. Proportion tests were used to identify overrepresented annotation results.

### Pathway Analysis

The *chipenrich* R Bioconductor package (version 2.6.1) was used to evaluate biological pathways enriched with significant DMRs (Welch et al., 2014). Four analyses were performed stratified by tissue and sex (i.e., male blood, male liver, female blood, and female liver). Locus definition *nearest_tss* (the region spanning the midpoints between the TSSs of adjacent genes) was used to discover enriched Gene Ontology (GO) terms. All three ontologies (i.e., Biological Process, Cellular Component, and Molecular Function) were used. An FDR < 0.05 cutoff was used for selecting significantly enriched GO terms.

### Annotating DMR Genes With Mouse Imprinted Genes and the CTD Database

To further interpret our results, we compared DMRs to mouse imprinted genes and the Comparative Toxicogenomics Database (CTD). Mouse imprinted genes were collected from Williamson et al. (2013) and Tucci et al. (2019). After removing redundancy, 303 genes were used for comparison. The genome annotation GTF file (NCBI *Mus musculus* Annotation Release 108) was used to obtain mouse non-imprinted genes (49,846 genes) and calculate the proportion of non-imprinted genes relate to DMRs. Imprinted genes annotated to DMRs were firstly identified for each tissue and sex, and then the overlaps determined. For CTD, all mouse genes with Pb-acetate exposure were downloaded from CTD. Genes with interaction of Pb-acetate on the mRNA level with changes in expression or methylation were extracted. Similar to above, genes annotated to DMRs from each tissue and sex were firstly compared to the CTD genes. We then combined these results to determine which genes were identified across tissues and sexes.

## Results

### Differential DNA Methylation (DMCs and DMRs) With Perinatal Pb Exposure

**FIGURE 1:**
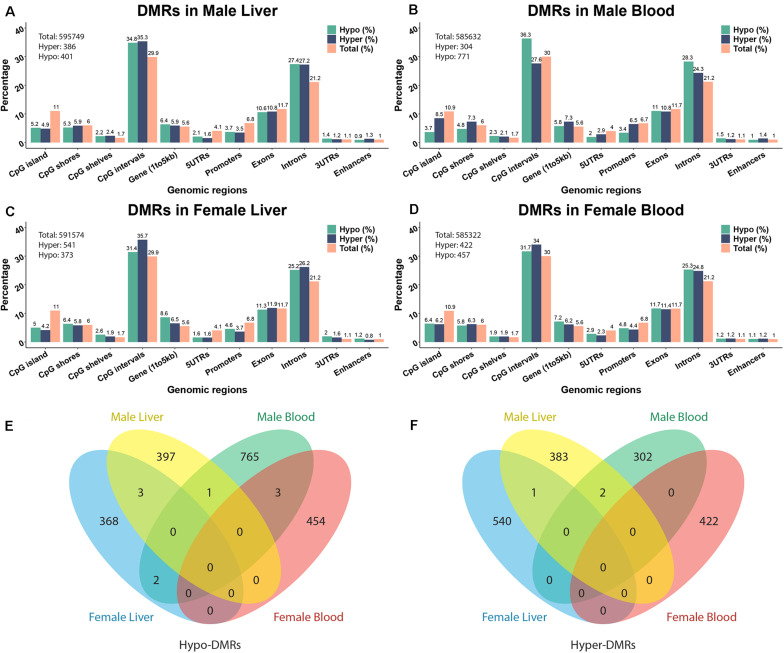
The annotation of Pb-associated differentially methylated regions (DMRs) and related genes. **(A–D)** depict DMR annotations of male liver, male blood, female liver, and female blood. **(E,F)** Venn diagram showing the overlapping of hypo- and hyper-DMRs from different tissues in two sexes. The DNA methylation changes are expressed as lead vs control.

To investigate the effects of perinatal Pb exposure on DNA methylation, we performed ERRBS on liver and blood DNA from offspring mice on post-natal day 21 (PND21). The numbers of total examined cytosines/regions were consistent across tissues and sexes (see Figure 1 and Supplementary Table S1). From our ERRBS data, for both tissues, about 5% of all CpG sites across the mouse genome were tested to identify statistically significant DMCs and DMRs. Genome-wide sample correlations of CpG methylation indicates the samples were more highly correlated within groups (see Supplementary Figure S1). The average bisulfite conversion rate and read depth of all covered CpG sites from our ERRBS samples were 99.9% and 93.2, respectively (see Supplementary Table S2). About 1000 significant DMCs and hundreds of significant DMRs were detected by comparing Pb treated and control mice from each sex and tissue combination. The methylation changes of most cytosines/regions were 10 to 30%, with some as high as 67% (see Supplementary Figure S2 for distribution). Most changes were tissue and sex specific. From the results, we found male blood contained the most DMCs/DMRs compared to the others, with the majority being hypo-methylation. In other sex-tissue combinations, similar numbers of hypo- and hyper-DMCs/DMRs were identified. Among the DMRs, 1 hypo-DMR and 2 hyper-DMRs were identified in both male tissues. However, no DMRs were discovered in both female tissues. In liver, 3 hypo-DMRs and 1 hyper-DMR were found across sexes, while in blood, 3 hypo-DMRs were found for both sexes (Figure 1E). A similar number of overlapping DMCs were identified (see Supplementary Figure S3 for DMC Venn diagram).

The significant hyper- and hypo-DMRs were annotated to examine their genomic locations relative to all tested CpG sites and regions (Figure 1). The genomic locations of these DMRs revealed a similar pattern across tissues and sex. From the proportion test, most of the CpG sites/regions are significantly enriched in different genomic regions including CpG islands, 5′UTR, promoter region, intron, etc. (Supplementary Table S3). Although hundreds of genes are related to DMRs, only a small number of common genes are identified across tissues and sexes (see Supplementary Figure S4). 38 and 29 genes were identified across tissues in male and female, respectively. Five genes were detected in both tissues and in both sexes, i.e., *Prdm16*, *Hjurp*, *Cdh23*, *Bc1*, and *Arid1b*. Associated pie charts show the percentage of the genes related to hyper-, hypo-, or mixed (i.e., hyper and hypo) direction DMRs. From these pie charts, only a small number of genes are associated with mixed direction DMRs (see detail in Supplementary Table S4).

### Pathway Analysis of Tissue- and Sex-Specific DMRs

**FIGURE 2:**
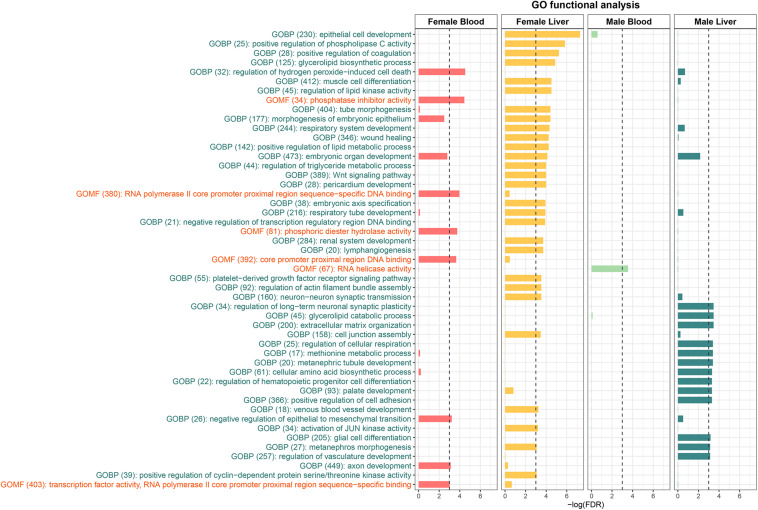
The Enriched GO terms identified from the DMRs. GOBP stands for Gene Ontology Biological Process and GOMF stands for Gene Ontology Molecular Function. No Gene Ontology Cellular Component terms were significantly enriched.

To further understand the biological pathways altered by perinatal Pb exposure, we investigated the enrichment of cellular pathways with DMRs. Significant DMRs from each tissue- and sex-specific analysis were used for enrichment testing, and FDR < 0.05 was used to select significantly enriched GO terms. Before removing redundant identified GO terms, three common GO terms *metanephros morphogenesis*, *sensory perception of chemical stimulus*, and *metanephric nephron morphogenesis* were discovered in liver across sexes. In the non-redundant results, female liver had the most enriched terms, while male blood only had one GO term (GO:0003724) (Figure 2). To further investigate the DMRs behind these enriched GO terms, we found most GO terms are enriched by mixed DMRs (i.e., hyper and hypo-methylated) (see Supplementary Table S5). However, in female blood, there is one GO term (GO:1903205) is enriched only by hyper-DMRs. In female liver, there is an enriched GO term (GO:0045737) that only related to hypo-DMRs. In addition, four GO terms (GO:0043457; GO:0072170; GO:1901532; GO:0003338) are enriched only by hypo-DMRs in male liver.

### Several Mouse Imprinted Genes Contain DMRs

The effects of perinatal Pb exposure on imprinted genes in liver and blood were investigated. In total, 1879 genes were related to DMRs from our results (Supplementary Figure S4). Overall, 303 mouse imprinted genes were used for this comparison, with 36 genes (11.88%) being associated with significant DMRs (Table 1). This is compared to 3.69% of all other genes (1843 of 49,846 total genes) associated with significant DMRs. Within these 36 genes, *Arid1b* contained DMRs in both liver and blood across both sexes. Besides *Arid1b*, no imprinted genes with DMRs were detected in liver across sexes. In blood, the imprinted gene *Trappc9* had DMRs in both sexes. In female liver and blood, *Trappc9* and *Smoc2* both had DMRs. In male liver and blood, besides *Arid1b*, *Pde10a* contained DMRs across tissues (see Supplementary Table S6 for details).

**TABLE 1:** Imprinted genes with differentially methylated regions (DMRs).

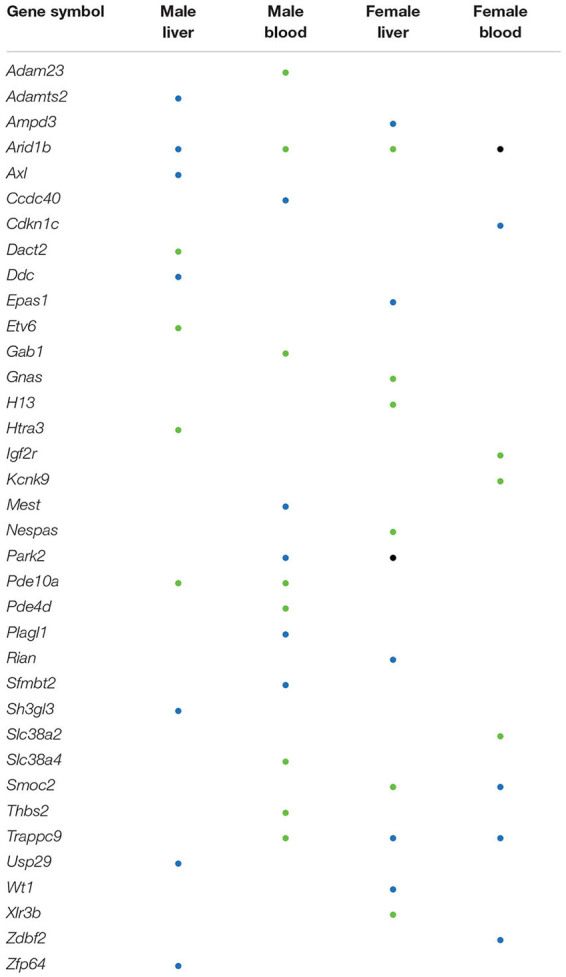

*The colors of dots indicate the types of DMRs that associated with the genes. Blue represents hyper-DMRs, green represents hypo-DMRs, and black represents mixed-type (hyper and hypo) DMRs.*

### Pb-Associated Genes in the Comparative Toxicogenomics Database Contain DMRs

**FIGURE 3:**
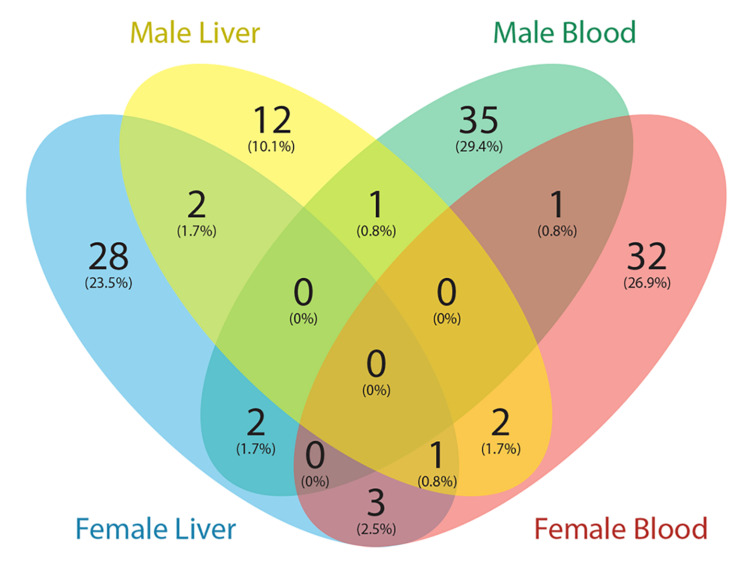
Venn diagram showing the number of genes with DMRs overlapped with Pb exposure related genes from the Comparative Toxicogenomics Database (CTD).

The Comparative Toxicogenomics Database (CTD) (Mattingly et al., 2006) is a curated resource that annotated known mouse Pb exposure-related target genes. Genes with expression changes and methylation changes due to Pb exposure were collected from the CTD (data updated March 2020), and after removing redundancy, 1190 genes were identified. Of these 1190 genes, only one gene contained a DNA methylation change in an exon region according to the CTD. Therefore, we focused on genes with Pb exposure related expression changes in the CTD. In total, 119 genes with at least one DMR from our results were annotated in CTD with changes at the transcription level due to Pb exposure. These 119 genes are therefore likely to be dysregulated at least partially due to DNA methylation changes in response to Pb. In female tissues, 39 and 36 genes with DMRs from blood and liver were Pb-associated, respectively. *Art3*, *Bri3*, *Ephb1*, and *Ldlrad4* were the four genes found across female tissues. In male tissues, 39 genes were detected from blood and 18 genes were detected from liver. *Prkca* was the only gene detected across male tissues (Figure 3). In blood, 9 and 8 genes were involved in nucleic acid-templated transcription in female and male, respectively. In male liver, there were six genes in this biological process. Gene list and associated DMRs can be found in Supplementary Table S7.

## Discussion

Pb is a common environmental exposure that is related to many health problems. In this study, we specifically investigated the effect of perinatal Pb exposure on DNA methylation in two tissues and both sexes using an established mouse model of perinatal environmental exposures. Because regulated DNA methylation targets are usually clustered into certain regions, and not single CpG sites, we focused on analysing DMRs to explore the biological effects of the Pb-induced methylation changes (Gaspar and Hart, 2017). In the two female tissues as well as in the male liver, similar numbers of hyper- and hypo-DMRs were identified. In male blood, however, we observed many more hypo-methylated than hyper-methylated regions. The sequencing depth of male blood samples did not show any bias compared to other tissues, suggesting that the result is not due to a technical effect of coverage level. However, it is possible that the trend toward hypomethylation is due to an unknown technical effect. If biologically meaningful, the biological mechanism underlying this observation is currently unclear. A potential explanation is that perinatal Pb exposure can modulate the expression of *Dnmt1* and reduce the activity of DNMT1 (Wu et al., 2008; Schneider et al., 2013; Ordemann and Austin, 2016), the maintenance DNA methyltransferase (Sobolewski et al., 2018); thus, sex- and tissue-specific alterations in *Dnmt1* expression may contribute to the high level of hypo-methylation in male blood. Future mechanistic studies are necessary to answer this question.

Previous studies revealed that DNA methylation in different genomic contexts relates to unique biological functions. The DNA methylation status of non-CpG island sites are more dynamic and tissue-specific than CpG islands (Jones, 2012). After annotating DMRs to genic regions, we found five genes - *Prdm16*, *Hjurp*, *Cdh23*, *Bc1*, and *Arid1b* – that were detected with DMRs across a combination of tissues and sex. Among these genes, four of them displayed DMRs mostly within intronic regions with mixed methylation directions (i.e., hyper and hypo). *Hjurp* showed inconsistent methylation direction within each tissue (hypo- in blood and hyper- in liver) across sex on an exon/3′UTR region. The gene products of *Hjurp* have been identified as an important factor of DNA binding that promotes cell mitosis and chromosomal segregation (Barnhart-Dailey et al., 2017). This gene has been linked to many cancers including liver cancer, breast cancer, lung cancer, and glioma (Chen et al., 2018). Previous work suggests that methylation in exons can affect the alternative splicing events of the gene (Shayevitch et al., 2018), so perinatal Pb exposure may affect the function of *Hjurp* on a transcriptional regulation level through alternative splicing caused by DNA methylation changes.

In liver, *Prdx6b* was identified with a hyper-methylation change on an exon region in male and hypo-methylation in female. *Prdx6b* is an antioxidant gene involved in redox regulation of the cell. It has been reported that an elevated blood Pb concentration is associated with an increased risk of non-alcoholic fatty liver disease (Zhai et al., 2017). Mice with highly expressed *Prdx6b* in liver showed more protection from lipid accumulation after a high-fat diet (Lee et al., 2019).

In blood, we found the promoter region of *Chchd2* is hypo-methylated in both sexes. This gene is associated with Parkinson’s disease in humans, which is a long-term degenerative disorder of the central nervous system (Funayama et al., 2015). Our results show that perinatal Pb exposure can affect the methylation status of the promoter region of this gene in a surrogate tissue at a young age (PND21) in mouse. The biological consequences of the altered DNA methylation resulting from perinatal Pb exposure need to be further examined.

To further understand the biological pathways associated with DNA methylation changes caused by perinatal Pb exposure, we identified GO terms enriched with identified DMRs. In female liver, GO terms *triglyceride metabolic process*, *lipid metabolic process*, and *regulation of lipid kinase activity* were significantly enriched. Similar GO terms *methionine metabolic process* and *glycerolipid catabolic process* were enriched in male liver. Previous studies show Pb exposure can damage the cell membranes through lipid peroxidation in liver (Sandhir and Gill, 1995). As a target tissue of Pb exposure, liver plays an important role in fat metabolism. From this study, the results suggest the perinatal Pb exposure can affect metabolic pathways through the alteration of DNA methylation. In female blood, *regulation of hydrogen peroxide-induced cell death* and *phosphatase inhibitor activity* were the most highly enriched. However, in male liver, the same GO terms are not significantly enriched. In blood, only a handful of GO terms were enriched, but it also shows more enriched GO terms in females than males. These together indicate that there are sex differences in the effects of perinatal Pb exposure.

Genomic imprinting is an epigenetic phenomenon in which the mono-allelic expression of certain genes occurs in a parent-of-origin specific manner. This phenomenon is caused by differential epigenetic modifications that are established in the germline and maintained through mitotic cell division in next generation (Tucci et al., 2019). Genomic imprinting is critical to normal development, and defects in imprinting are associated with neurodevelopmental and metabolic diseases (Bartolomei and Ferguson-Smith, 2011). Recent studies demonstrate that genomic imprinting is sensitive to environmental exposures as well as maternal nutrition and metabolic status (Monk et al., 2019). Imprinted genes are typically controlled by imprinting control regions (ICRs) and identification of ICRs is currently an active research area. Existing studies indicate that the ICR can vary by developmental stage and tissue. For example, the expression of *Gnas* in the mouse blood can be affected by DNA methylation changes that are not located in the previous identified imprinting control region (Kochmanski et al., 2018; Svoboda et al., 2019). The predefined ICR of this gene is within exon 1A of the *Nespas* gene, however, the DMR detected from our data did not overlap with this ICR. It is plausible that the DMRs located on non-ICRs may still be important for regulation of gene expression.

From our results, one imprinted gene, i.e., *Arid1b*, was differentially methylated across both sexes and tissues. The product of this gene is a component of chromatin remodeling complex and may also play a role in cell cycle activation (Li et al., 2010; Santen et al., 2012). This gene contained DMRs in intronic regions in all four comparisons, thus the regulation of this gene through DNA methylation is worth further examination. In females, *Smoc2* was annotated to hyper-DMRs and hypo-DMRs in blood and liver, respectively. This gene is related to calcium ion binding and glycosaminoglycan binding (Peeters et al., 2016). In our results, the 3′UTR of this gene was covered by hyper-DMRs and hypo-DMRs in blood and liver, respectively. Since the 3′UTR is a putative functional DNA methylation site that can affect the expression of genes (Choi et al., 2009; McGuire et al., 2019), further studies are needed to address the potential functional consequences of altered methylation of this gene in female tissues. Overall, the percentage of imprinted genes with DMRs (11.88%) is higher than other genes with DMRs (3.69%). This suggests that perinatal exposures might differentially affect the methylation status of imprinted genes and further impact the normal developmental process.

To further investigate our results, we compared genes with DMRs from this study to those linked to Pb exposure in the Comparative Toxicogenomics database (CTD). We downloaded all mouse Pb exposure related target genes from the CTD to perform the analysis, and found a small portion (∼10%) of genes from the CTD were identified with DMRs in this study. Since the CTD dataset mainly represents genes that have an expression level change with Pb exposure, and our study was focused on DNA methylation changes, the limited overlap between these two datasets is not surprising. Among these genes, *Prkca* is the only gene identified in both male tissues (hypomethylation of 3′UTR in blood and intronic hypermethylation in liver). The product of this gene, protein kinase C (PKC), is strongly activated by Pb, causing dysregulation of neurotransmitter release and second-messenger systems (Tarrago and Brown, 2017). Our results indicate that this gene can be epigenetically affected by Pb exposure in blood and liver. In the CTD database, expression of *Prkca* was affected by Pb exposure at both the mRNA and protein levels. Thus, the methylation changes of this gene may contribute to the observed expression changes.

Since ERRBS was used to examine the DNA methylation changes and only about 10% of total CpG sites in mouse genome were covered by solid reads, this study cannot provide the whole genome-wide DNA methylation changes caused by perinatal Pb exposure. However, tissue and sex specific DMRs were still identified from our data. Future studies should focus on finding other potential perinatal Pb exposure related methylation changes, discovering the longitudinal biological consequences of the perinatal Pb exposure, and properly applying the findings to human environmental epigenetics studies.

## Data Availability Statement

All ERRBS data is publicly available at Gene Expression Omnibus (GEO) under accession ID GSE150670.

## Ethics Statement

The animal study was reviewed and approved by The University of Michigan Institutional Animal Care and Use Committee (IACUC).

## Author Contributions

MS, DD, LS, and JC conceived of the study. CR, TJ, and KN performed the tissue collection and sample preparation for ERRBS. KW and SL carried out the data analysis. KW wrote the manuscript and all authors edited and approved the manuscript.

## Conflict of Interest

The authors declare that the research was conducted in the absence of any commercial or financial relationships that could be construed as a potential conflict of interest.

## Supplementary Material

FIGURE S1 The genome-wide weighted sample correlations.

FIGURE S2 The distribution of DNA methylation changes of differentially methylated cytosines (DMCs) and differentially methylated regions (DMRs).

FIGURE S3 The overlap among differentially methylated cytosines (DMCs) among sexes and tissues.

FIGURE S4 Venn diagram showing the number of DMR related genes detected from different tissues in two sexes along with associated pie graph depicting the percentage of methylation directions of each gene set. The DNA methylation changes are expressed as lead vs control.
