# Supplementary figures and images for: Tissue- and Sex-Specific DNA Methylation Changes in Mice Perinatally Exposed to Lead (Pb)
Source: Front Genet. 2020 Aug 21;11:840. doi: 10.3389/fgene.2020.00840 (PMC7472839; doi:10.3389/fgene.2020.00840)

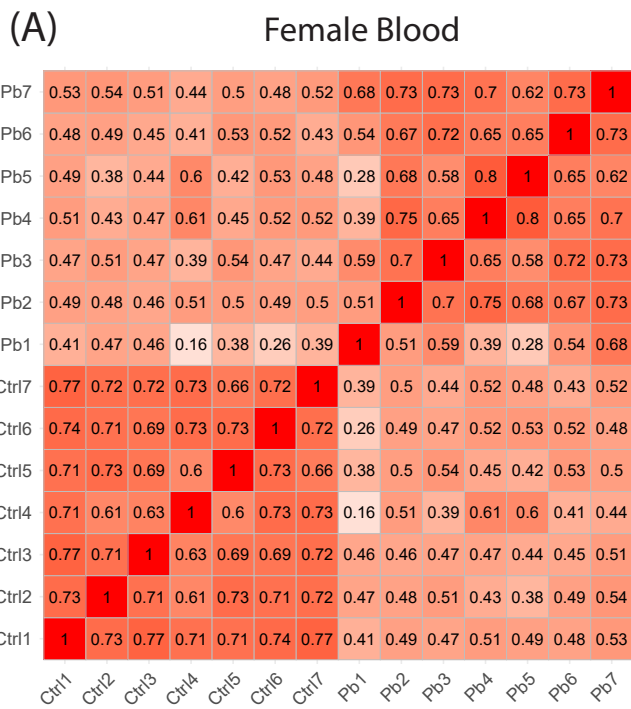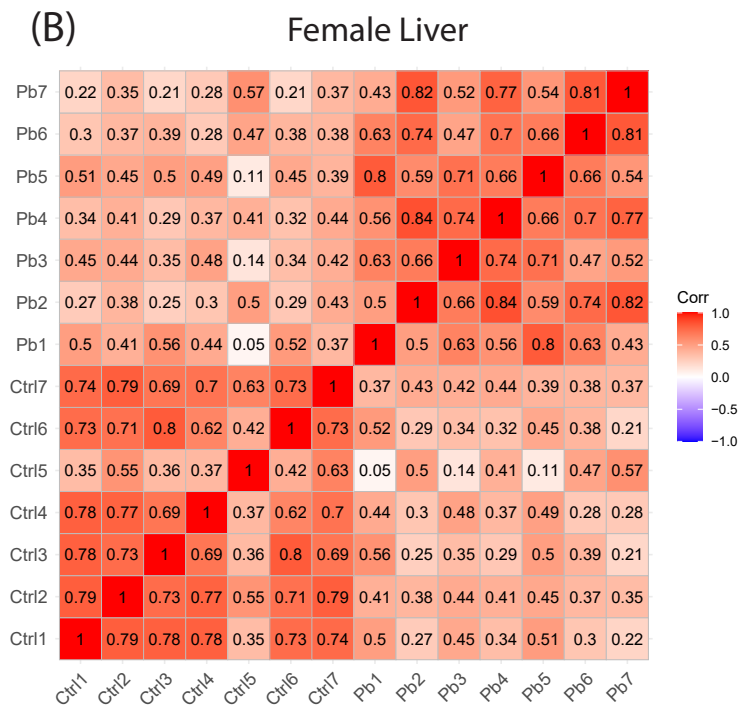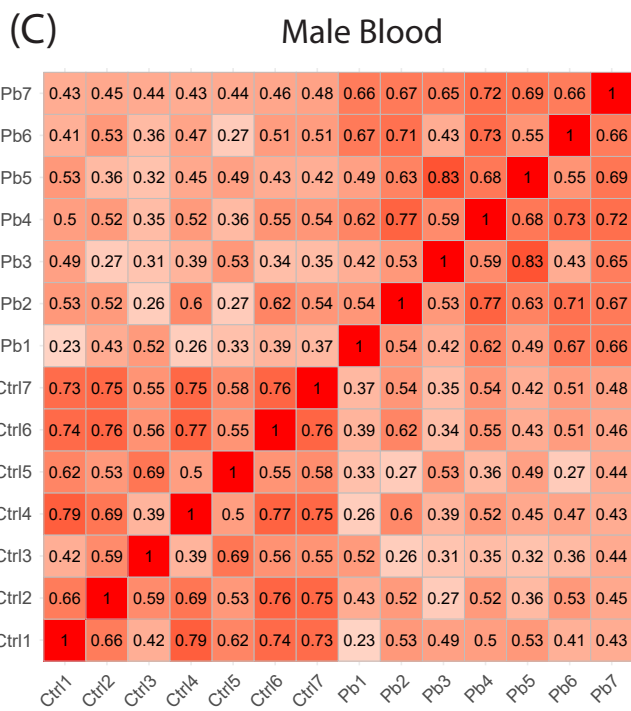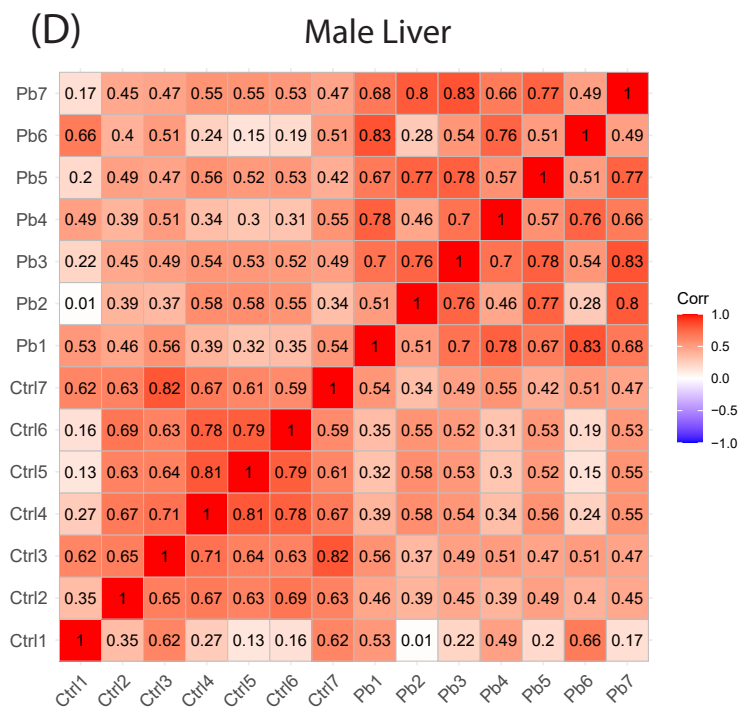

Supplement: FIGURE S1 — The genome-wide weighted sample correlations. [file Image_1.PDF]

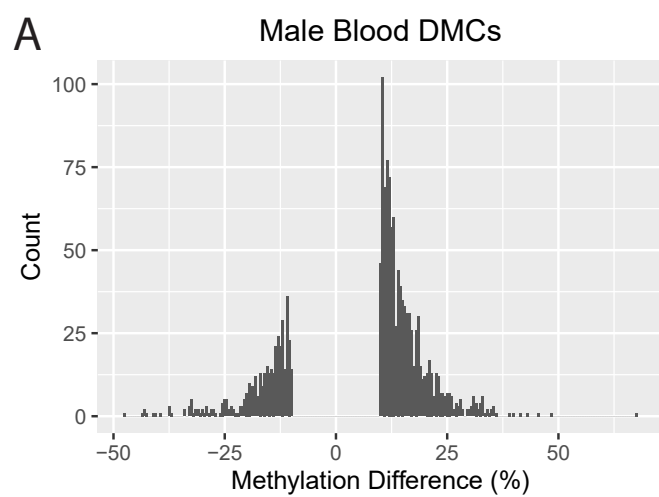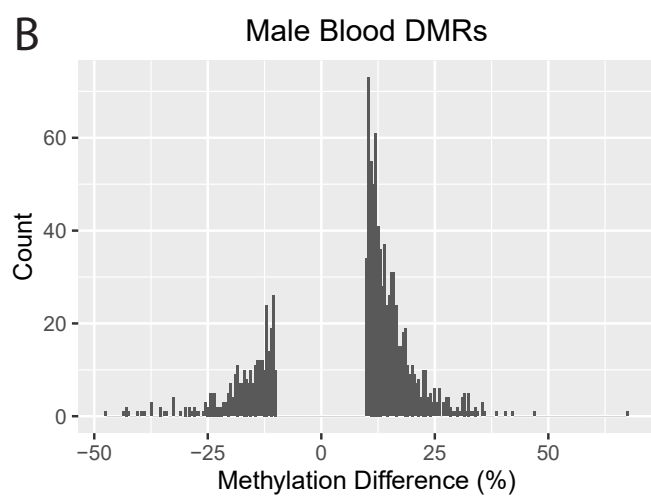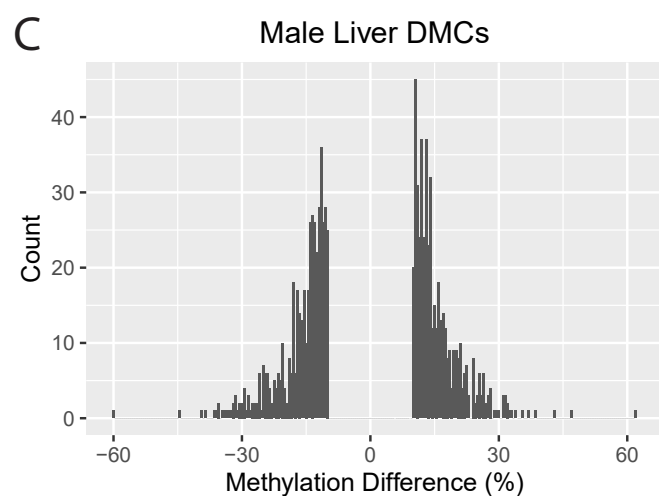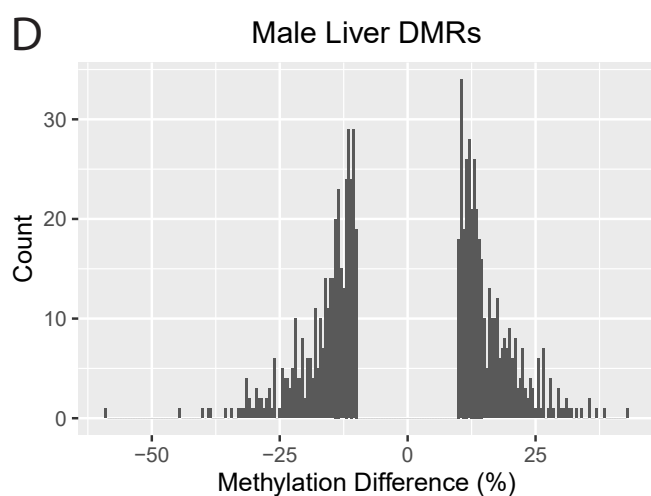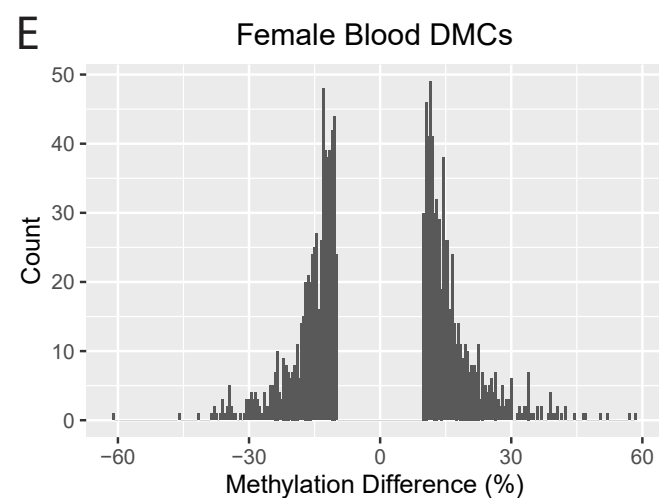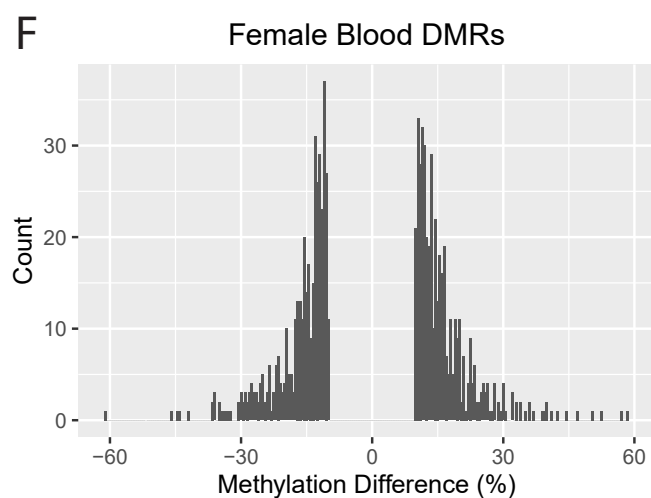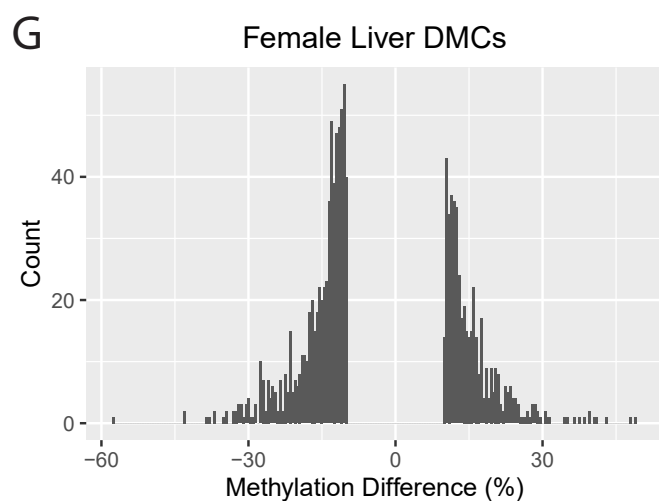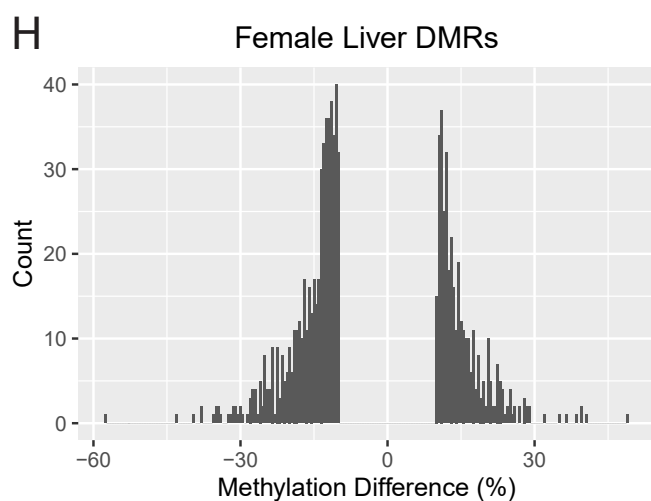

Supplement: FIGURE S2 — The distribution of DNA methylation changes of differentially methylated cytosines (DMCs) and differentially methylated regions (DMRs). [file Image_2.PDF]

(A)

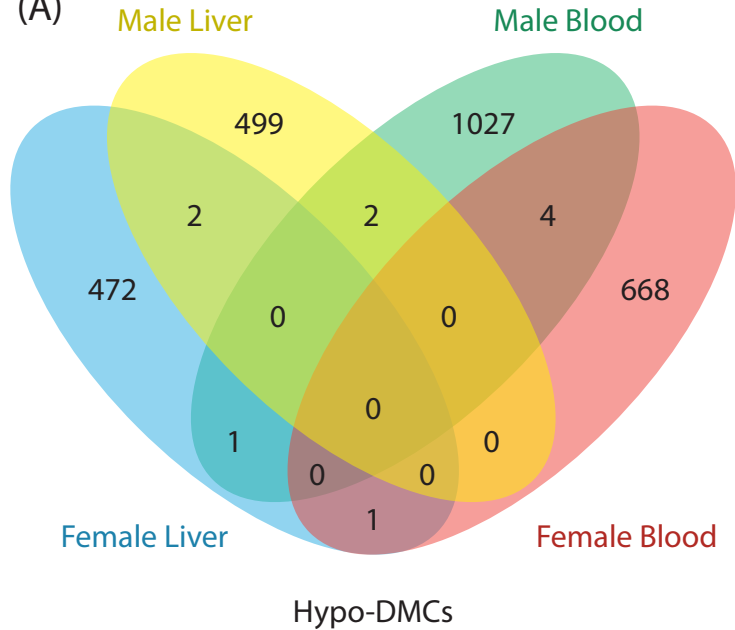

(B)

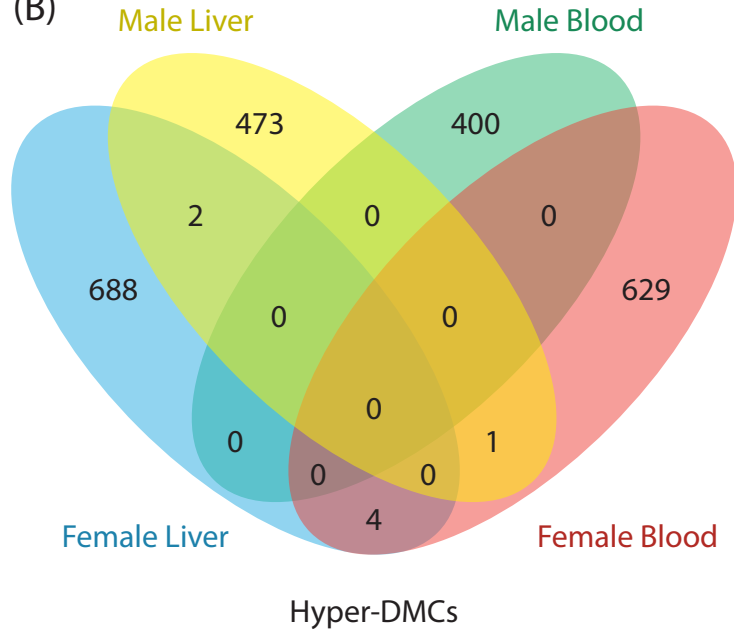

Supplement: FIGURE S3 — The overlap among differentially methylated cytosines (DMCs) among sexes and tissues. [file Image_3.PDF]

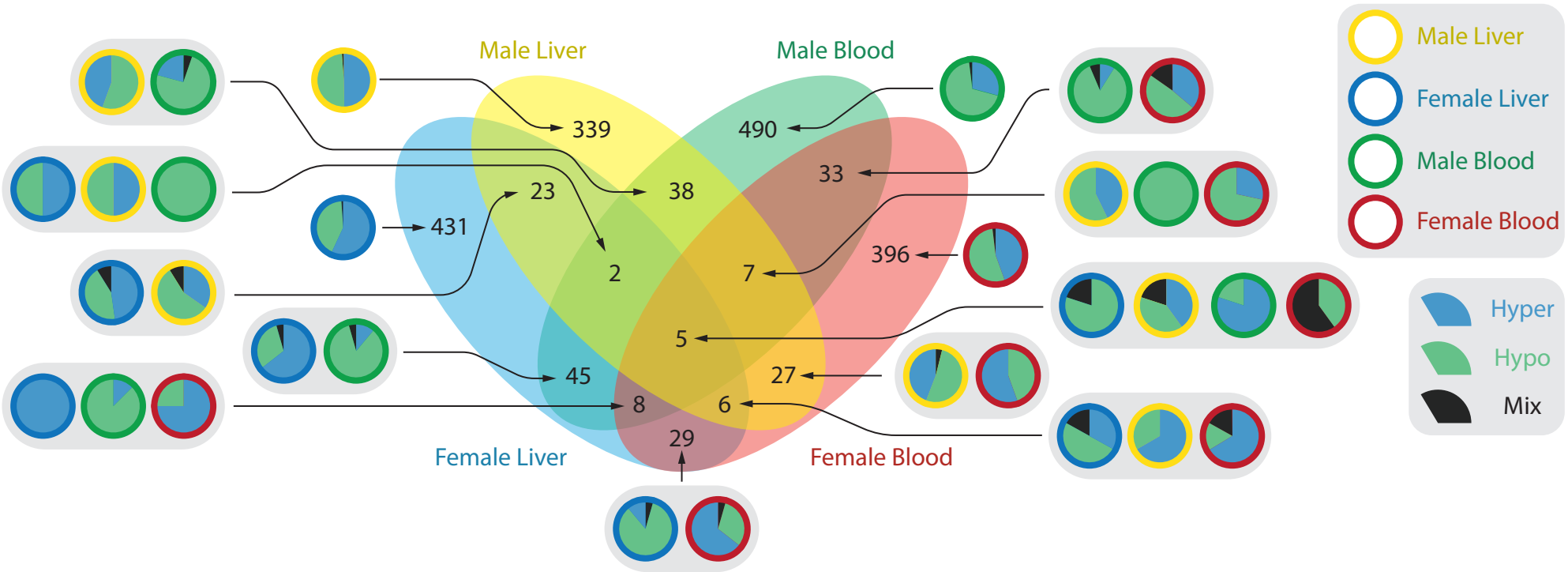

Supplement: FIGURE S4 — Venn diagram showing the number of DMR related genes detected from different tissues in two sexes along with associated pie graph depicting the percentage of methylation directions of each gene set. The DNA methylation changes are expressed as lead vs control. [file Image_4.PDF]
